# Supplementary material for: Iterative improvement in the automatic modular design of robot swarms
Source: PeerJ Comput Sci. 2020 Dec 7;6:e322. doi: 10.7717/peerj-cs.322 (PMC7924708; doi:10.7717/peerj-cs.322)
Supplement: Supplemental Information 3 [file peerj-cs-06-322-s003.zip › argos3/doc/api/standalone/a00513_source.html]

ARGoS: plugins/robots/generic/control\_interface/ci\_quadrotor\_speed\_actuator.h Source File


- Main Page
- Related Pages
- Namespaces
- Classes
- Files

- File List
- File Members

# plugins/robots/generic/control\_interface/ci\_quadrotor\_speed\_actuator.h

Go to the documentation of this file.

```
00001 
00007 #ifndef CI_QUADROTOR_SPEED_ACTUATOR_H
00008 #define CI_QUADROTOR_SPEED_ACTUATOR_H
00009 
00010 /* To avoid dependency problems when including */
00011 namespace argos {
00012    class CCI_QuadRotorSpeedActuator;
00013 }
00014 
00015 #include <argos3/core/control_interface/ci_actuator.h>
00016 #include <argos3/core/utility/math/vector3.h>
00017 
00018 namespace argos {
00019 
00020    class CCI_QuadRotorSpeedActuator : public CCI_Actuator {
00021 
00022    public:
00023 
00024       virtual ~CCI_QuadRotorSpeedActuator() {}
00025 
00030       virtual void SetLinearVelocity(const CVector3& c_velocity) = 0;
00031 
00036       virtual void SetRotationalSpeed(const CRadians& c_speed) = 0;
00037 
00038 #ifdef ARGOS_WITH_LUA
00039       virtual void CreateLuaState(lua_State* pt_lua_state);
00040 #endif
00041 
00042    };
00043 
00044 }
00045 
00046 #endif
```

---

Generated on 10 Jul 2018 for ARGoS by 
 1.6.1
